# Feasibility of a multifaceted nutrition-risk screening tool for mental health settings: the NutriMental screener

**DOI:** 10.1017/S0007114524002101

**Published:** 2026-03-14

**Authors:** Annabel S. Mueller-Stierlin, Sonja Mötteli, Florian Hotzy, Sabrina Mörkl, Tracy Burrows, Oliver Ardill-Young, Ramona Hiltensperger, Polona Rus Prelog, Scott B. Teasdale

**Affiliations:** 1 Institute for Epidemiology and Medical Biometry, Ulm University, Ulm 89070, Germany; 2 Center for Psychiatric Rehabilitation, Universitäre Psychiatrische Dienste Bern (UPD), Bern, Switzerland; 3 Department of Psychiatry, Psychotherapy and Psychosomatics, Psychiatric Hospital of the University of Zurich, Zurich 8032, Switzerland; 4 Klin. Abteilung für Med. Psychologie, Psychosomatik und Psychotherapie, Medzinische Universität Graz, Graz, Austria; 5 University of Newcastle, College Health Medicine and Wellbeing, Callaghan, NSW, Australia; 6 Discipline of Psychiatry and Mental Health, School of Clinical Medicine, UNSW Sydney, Sydney, NSW 2052, Australia; 7 Mindgardens Neuroscience Network, Sydney, NSW 2052, Australia; 8 Centre for Clinical Psychiatry, University Psychiatric Clinic Ljubljana, Ljubljana, Slovenia; 9 Medical Faculty Ljubljana, University of Ljubljana, Ljubljana, Slovenia

**Keywords:** Diet, Nutrition, Mental health, Mental illness, Risk screening, Nutritional psychiatry

## Abstract

People living with mental illness report a broad spectrum of nutrition risks, beyond malnutrition, but appropriate and adequately validated nutrition risk screening tools for mental health settings are lacking. This study aimed to develop a nutrition-risk screening tool, the NutriMental Screener, and to perform preliminary feasibility and validity testing. In an international, stakeholder engaging approach, a multifaceted nutrition-risk screening tool for mental health services was developed by means of workshops with international stakeholders and two online surveys. Feasibility of the NutriMental screener was tested as part of a research study in Switzerland with 196 participants, evenly distributed across the three study groups (sixty-seven outpatients and sixty-five inpatients with psychotic or depressive disorders as well as sixty-four controls without mental illness). The NutriMental screener consists of ten items covering different nutritional issues that indicate the need for referral to a dietitian or clinical nutritionist. Almost all patients (94·7 %) reported at least one nutrition risk by means of the NutriMental screener. Prevalence for nutrition risks via NutriMental screener was higher in patients than in controls. Almost every second patient expressed a desire for nutritional support (44·7 %). After further validity testing is completed, there is the potential for the NutriMental Screener to replace malnutrition screening tools as routine screening in various mental health settings aiming to organise nutritional therapy prescriptions in a more targeted and efficient manner.

The treatment offered to people with a mental illness is evolving to become more holistic, encompassing both mental and physical health and acknowledging the social determinants of health^(1,2)^. More recently, lifestyle-based interventions have been recommended as a foundational component of mental health care in clinical practice for people living with mood disorders by leading international medical societies^(3,4)^. As part of this, the role of nutrition in people with mental illness is becoming more defined; poor diet quality is a modifiable risk factor for mental illness and an adjunctive treatment^(5)^. In addition, the last few decades have seen a shift to managing physical health complications, such as metabolic syndrome, diabetes and CVD, which occur at disproportionate rates in people with mental illness. Nutrition intervention is considered a central element in preventing and managing the unacceptable rates of physical comorbidity, which often drastically reduces life expectancy for people with mental illness^(1,2)^.

This shift is leading to more nutrition professionals working in mental health settings^(6)^, and with this, a need to be able to identify people with nutrition risks and refer to these specialist clinicians. Traditional nutrition-risk screening tools used in other hospital settings typically focus on undernutrition given the longer hospital stays, higher health costs and poorer health outcomes linked to undernutrition^(6)^. Inpatients from non-mental health services are frequently recovering from illness or injury that requires greater nutritional needs, while potentially having a reduced appetite. For people receiving support from mental health services, the needs and priorities can differ. While undernutrition remains important^(7)^, many experience substantial appetite and food preference changes^(8,9)^, disordered eating^(10–12)^ and food insecurity including barriers to food access, storage and use^(13–15)^. This suggests a broader definition of nutrition risk is needed for use in mental health services.

A recent scoping review of nutrition screening tools used in mental health settings found a dearth of appropriate and adequately validated tools^(16)^. The seventeen tools identified had restricted foci: one focused solely on malnutrition, one focused solely on dysphagia, three assessed constipation only, eleven assessed disordered eating only and one was a checklist of medication side-effects. Additionally, only three of these tools had been validated in mental health settings^(16)^.

The scoping review was used as a starting point for a five-phase process to develop a targeted nutrition-risk screening tool for mental health services (see Fig. 1). In phase 1, the lead authors obtained all screening tools described in the scoping review by Hancox et al.^(16)^ and inductively coded each of the 196 individual questions/items in a consensual procedure in order to develop six overarching domains and twenty-six themes of interests that have been used in nutrition screening tools in mental health settings^(17)^. In phase 2, we then conducted additional background work to inform the development of a draft tool, this included literature reviews on dietary intake^(9,18)^, eating behaviour^(10,11,19)^ and rates and correlates of food insecurity experienced by people with severe mental illness^(13)^. In addition, we conducted semi-structured interviews with mental health service users in Australia, Austria and Germany^(12,20)^. Taken together, a broad scope of nutrition-related challenges faced by people with mental illness was revealed, such as physical comorbidities, changes in weight and appetite, disordered eating and food insecurity.

**Fig. 1.**
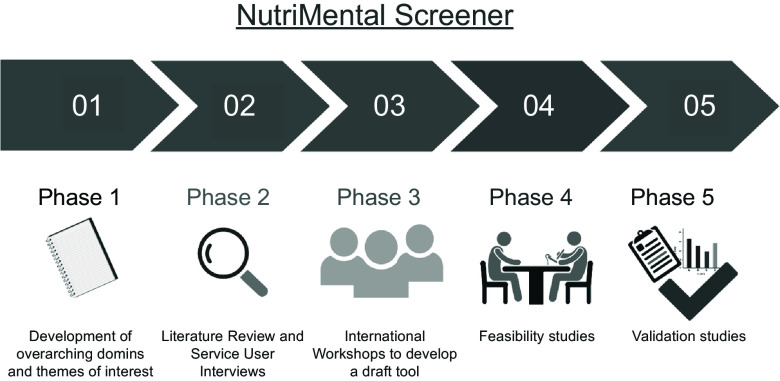
Development and validation phases of the NutriMental Screener tool. This figure was created with biorender.com (accessed on 21 March 2024).

Co-creation with service users, health professionals and researchers is more likely to generate outcomes that can be implemented in real-world settings than developing tools by scientists alone^(21)^. Accordingly, stakeholder engagement was emphasised in the subsequent phases of the development process. This study aimed (i) to develop a draft nutrition-risk screening tool, the NutriMental Screener, through a structured and participatory approach (phase 3) and (ii) to perform preliminary feasibility and validity testing (phase 4).

## Materials and methods

### Development of a draft nutrition-risk screening tool (phase 3)

#### Design

A modified Delphi approach was applied in a three-stage process that included: stage 1) an online workshop to determine the objective of the tool with a subsequent online survey, stage 2) item selection and drafting the tool and stage 3) an online survey with subsequent online workshop to determine relevance and wording of the items^(22)^.

#### Setting and participants

Participants were a multi-national working group of mental health clinicians, dietitians and peer workers. Participants were recruited through the authors’ personal networks.

#### Procedure

The three-stage process was led by authors AMS and ST and commenced with an online workshop via Zoom which was followed by two online surveys with implied consent via SoSci Survey and finished with a second online workshop via Zoom.

Stage 1: In workshop 1 (June 2021), the rationale for the tool development and overall design were presented to the working group. Consensus was sought from workshop participants on the objective of the tool. Participants were then asked to rate anonymously the relevance for each of the six domains and each of the twenty-six themes (coded from Phases 1 & 2) on a five-point Likert scale from ‘1 = not relevant’ to ‘5 = very relevant’ during Workshop 1.

Stage 2: Based on the findings from the first workshop, AMS and ST selected potential items from the 194-item list (developed in Phase 1) for these most relevant themes (with a mean score > 4), adjusted them through discourse and drafted a first version of the NutriMental screener (see online Supplementary file 1).

Stage 3: Another online survey was then sent to the working group to rate anonymously the relevance of each item on a five-point Likert scale from ‘1 = not relevant’ to ‘5 = very relevant’, with an option for additional comments on relevance and wording in free text fields for each question/item. The findings from this survey were presented to Workshop 2 participants (July 2021) and discussions followed within the working group until consensus on inclusion and wording of each item was reached. An information sheet with short instructions in English for the use of the NutriMental Screener was created based on the discussions at the international workshops.

#### Analysis

Descriptive statistics were calculated for all survey ratings by means of SPSS Statistics Version 29 and presented as mean scores or absolute and relative frequencies.

### Feasibility (phase 4)

#### Design

Feasibility testing was embedded within a larger prospective study on nutritional needs in mental health care conducted in Switzerland between September 2021 and August 2022^(23,24)^. All participants provided informed consent. The feasibility testing was approved by the Swiss Ethics Committee, Switzerland (2019-01485). Reporting was completed according to the STROBE guidelines^(25)^.

#### Participants and setting

Participants were people aged 18–65 years living in the Zurich area, who spoke fluent German, and fitted into one of the following groups: (1) inpatients with psychotic or depressive disorders (International Classification of Disease codes: F2 or F3), (2) outpatients with psychotic or depressive disorders or (3) healthy controls (absence of a mental disorder). People with a primary or secondary diagnosis of an eating disorder were excluded. Inpatients and outpatients were receiving support from the Psychiatric Hospital of the University of Zurich (Psychiatrische Universitaetsklinik Zuerich). Stratified sampling for age and gender was used. Convenience sampling was used to recruit healthy controls, with strategies including flyers in family practice centres, shops and city swimming pools; university mailing lists and word-of-mouth by mental health professionals. Further details on the stratified sampling approach to recruitment are available in the protocol^(23)^.

A sample size calculation was conducted for the primary study^(23)^, using G * Power 3.1^(26)^. A total of 192 participants were needed to detect a between-group difference (*α* = 0·05) in nutrition knowledge including two covariates (education and previous nutrition counseling) using an ANCOVA assuming an effect size of *F* = 0·25 with 80 % power.

#### Procedure

Participants were interviewed by research assistants as part of the broader study at the Psychiatrische Universitaetsklinik Zuerich (both inpatients and outpatients) or at a location of their choosing (control group). Research assistants received training for standardised assessment of measures.

Interviews lasted approximately 1·5 h, with the participants able to pause the interview or complete over two sessions. Participants were offered a drink of their choice but did not receive financial reimbursement. Research assistants entered all assessment data into LimeSurvey following completion of the interview.

#### Outcome measures

The primary outcome measure for this study was the NutriMental screener. Following the development of the draft NutriMental Screener in English (described above), the tool underwent forward–backward translation to German with a concluding harmonisation conference between the first-language English investigator (ST) and first-language German investigators (AMS, SoM and SaM). The NutriMental screener was assessed for responses to individual questions and the total ‘yes’ responses to questions 1–9 summed for a total score from out of nine. We assumed that a higher NutriMental Score might indicate the need for a referral to a dietitian or clinical nutritionist. Item 10, participant desire for nutrition support, was assessed on its own as not considered a nutrition risk. A feedback questionnaire on the general feasibility of the NutriMental screener was completed by the five research assistants at the end of the 12-month recruitment phase. The purpose-built twelve-item feedback questionnaire included general ratings, ratings on acceptability, content, delivery/practicability, usefulness of instructions and effectiveness as well as open questions on satisfaction and suggestions for revisions.

Additional nutrition-risk screening tools were adapted versions of the Nutritional Risk Screening (NRS)^(27)^ and the Mini Nutritional Assessment-Short Form (MNA-SF)^(28,29)^. The NRS is a validated measure of malnutrition used in hospital settings. The NRS contains two components: nutritional status based on weight loss/BMI (0–3 points) and the severity of disease based on a categorisation of physical illnesses (0–3 points) for a total possible score of 6 points^(27)^. The latter (severity of disease) was measured in the adapted version by the modified Global Assessment of Functioning Scale^(30)^. A score of 3 or more indicates a risk for, or the presence of, malnutrition. The MNA-SF is also a screener for malnutrition used in hospital settings^(31)^. The MNA-SF includes a change in appetite (decrease), problems with swallowing and digestion, BMI, weight loss, mobility, psychological distress and mental conditions. The adapted version for this target group consisted of the following: (i) increase in food intake was included, (ii) weight-gain was included, (iii) mobility was broadened from physical problems to physical mobility in general, (iv) questions on stress and mental conditions were replaced with the modified Global Assessment of Functioning Scale ratings^(30)^ and (v) BMI categorisation was modified to include categories related to obesity. A score of 11 or less on the MNA-SF indicates a risk for, or presence of, malnutrition.

BMI was the key anthropometric measure related to this study and was calculated as weight (kg)/height (m)^2^. Weight was measured to the nearest 0·1 kilogram, using a digital scale (SOEHNLE 63 850 PWD Style Sense Compact 100). Height was measured to the nearest centimeter using a non-expandable SECA measuring tape. Each measurement was conducted twice, with the mean value being used.

In addition, we collected data on the demographic variables including age (years), gender (categorical) and education level (binary variable; compulsory education achieved).

#### Analysis

All analyses were performed using SPSS Statistics Version 29. Descriptive statistics were used for demographic details. Age and BMI were reported as mean and sd. Gender, International Classification of Disease code (F2 or F3) and highest education obtained were reported as number of people and percent of total group. Independent samples *t* tests and *χ* ^2^ tests were used to detect differences in continuous and categorical variables, respectively, between inpatients and outpatients as a combined group and controls to a significance level of *α* = 0·05. Responses to the NutriMental Screener were presented as number of people and percent responding ‘yes’ to each item within each group. *χ* ^2^ or Fisher exact tests were used to detect between group differences in responses between inpatients and outpatients as a combined group and controls. ANOVA was used to detect between group difference in mean NutriMental score between inpatients, outpatients and controls. In addition, independent samples *t* tests were used to test for a difference in mean NutriMental Scores for those patients who were at-risk as identified by the NRS and MNA-SF compared with those not at-risk, as well as, comparing mean NutriMental Scores for those who had a desire for dietary support compared with those who did not want dietary support. *χ* ^2^ tests were used to explore associations between individual items of the NutriMental screener and the desire for dietary support to a significance level of α = 0·10. The feedback questionnaire data were analysed in a narrative way.

## Results

### Development of the NutriMental screener

Twelve participants (six dietitians, four psychiatrists, one nurse and one peer support worker) from Australia, Austria, Germany, Slovenia and Switzerland engaged in Workshop 1. There was consensus from participants that the objective of the tool is to serve as a brief screener which flags nutrition-risks and leads to a more in-depth assessment and intervention where needed, ideally via referral to a dietitian/clinical nutritionist. According to the average relevance rating in the first online survey, the six clusters were ranked as follows: (1) eating behaviour (m = 4·64) and treatment (m = 4·64), (2) body (m = 4·45), (3) emotions (m = 4·36), (4) health state (m = 4·18) and (5) lifestyle (m = 4·00). For eight themes, the mean rating was above 4 points: weight change (body, m = 4·73), appetite and overeating (eating behaviour, m = 4·64), medication (treatment, m = 4·55), loss of control eating (eating behaviour, m = 4·36), appetite and malnutrition (eating behaviour, m = 4·20), emotional eating (emotions, m = 4·18), bowel habits (health state, m = 4·18) as well as craving and food preoccupation (eating behaviour, m = 4·09). Moreover, metabolic issues and food insecurity were mentioned as very relevant themes to be included in the screening tool (see Table 1).

**Table 1.** Mean relevance ratings (1 = not relevant, 5 = very relevant) for the six domains and 26 themes by 11 online participants at first workshop

Domain *or* theme (rating of domain and themes occurred independently)	Mean relevance rating
Risk factors related to disordered eating behaviour	4·64
Food preoccupation	3·64
Little appetite (undernutrition)	4·20
Much appetite (overnutrition)	4·64
Speed of eating	3·18
Loss of control eating	4·36
Food craving	4·09
Night eating	3·64
Eating structure	3·82
Food preferences	3·09
Risk factors related to eating behaviour and connected emotions	4·36
Eating shame/guilt	3·73
Emotional eating	4·18
Eating for a positive outcome	3·27
Risk factors related to body weight/shape and connected emotions	4·45
Weight change	4·73
Thinness	3·45
Weight preoccupation	3·45
Body dissatisfaction	3·55
Risk factors related to general health state	4·18
Memory/concentration	3·36
Swallow ability	3·91
Dry mouth/hypersalivation	3·64
Bowel habits	4·18
Other bodily issues	3·09
Risk factors related to the treatment of mental disorder	4·64
Medication	4·55
Risk factors related to lifestyle	4·00
Dieting	3·80
Compensatory behaviours	3·80
Exercise	3·18
Sleep hygiene	2·91

In the second online survey, ten out of fourteen items of the initial draft tool were rated as relevant or very relevant by all participants (6/6) or almost all (5/6) participants. There was a mixed voting pattern for the other four items (see Table 2).

**Table 2.** Proportion of participants, who rated the items of the first draft of the NutriMental Screener as relevant or very relevant in advance to the second workshop

	Item in initial draft	Proportion of participants who rated this item as relevant or very relevant	%
1	Are you/is the client underweight or overweight? {weight status}	5/6	83·3 %
2	Do you/does the client have any of the following conditions requiring dietitian/clinical nutritionist management? {clinical disorder}	6/6	100 %
3	Have you/has the client lost or gained more than 8 kg of weight without trying since taking the medication (or ‘over the last 6 months’)? {weight change}	6/6	100 %
4	Are you/is the client taking any of the following medications? {medication}	5/6	83·3 %
5	Since commencing the medication (or ‘In the last month’) have you/has the client had any significant changes in your/his/her appetite or eating behaviour? {appetite}	6/6	100 %
6	During the past month, have you/has the client felt you/they have frequently eaten what other people would regard as an unusually large amount of food given the circumstances? {disordered eating}	2/6	33·3 %
7	During the last month, have you/has the client frequently experienced strong urges to eat which you/they seem unable to control? {disordered eating}	5/6	83·3 %
8	In the last month, have there been several days when you/the client can’t seem to think about anything else but food? {disordered eating}	3/6	50·0 %
9	In the last month, has your/the client’s weight or shape negatively influenced how you/they think about yourself/themselves (judgement) as a person? {disordered eating}	2/6	33·3 %
10	During the past month, have you/has the client engaged in compensatory behaviours (e.g. fasting vomiting or taking laxatives) to prevent weight gain or counteract the effects of eating? {disordered eating}	5/6	83·3 %
11	Over the past month, have you/has the client had fewer than three defecations per week? {constipation}	6/6	100 %
12	In the last month, did you/the client worry that there would not be enough to eat because of lack of money? {food security}	3/6	50·0 %
13	Have you/has the client got access to specialist dietary support? {past dietary support}	5/6	83·3 %
14	Do you/does the client wish to be referred to the dietitian? {wish for dietary support}	5/6	83·3 %

As a result from the second workshop, items 6 and 9 were rated as not relevant and subsequently removed. The question about previous support (item 13) was removed through discussion, because it had no influence on whether further support is needed. Four items (item 7, 8, 10 and 11) were rephrased for better understanding. Items 1 and 2 were phrased more specific, e.g. by including a range for the BMI. The final tool is depicted in Table 3, and the German version is provided as an online Supplementary file.

**Table 3.** NutriMental screener data from feasibility study in research setting in Switzerland (Numbers and percentages; mean values and standard deviations)

		Outpatients (*n* 67)		Inpatients (*n* 65)		Controls (*n* 64)		Patients *v*. controls *P* value
Age (years)	m, sd	40·2	11·44	39·9	12·87	38·7	11·90	0·471
Female	*n*, %	34	50·7 %	32	49·2 %	32	50·0 %	1·000
BMI (kg/m^2^)	m, sd	27·9	5·30	25·3	4·98	24·0	3·70	< 0·001
ICD-10 F2x	*n*, %	9	13·4 %	25	38·5 %	0	0 %	< 0·001
ICD-10 F3x	*n*, %	58	86·6 %	40	61·5 %	0	0 %	< 0·001
Only compulsory education	*n*, %	9	13·4 %	14	21·5 %	2	3·1 %	0·001

### Feasibility testing of the NutriMental screener

#### Participant recruitment

Out of 637 potentially eligible patients from September 2021 to August 2022 at the Psychiatric Hospital of the University of Zurich (inpatients *n* 521, outpatients *n* 116), 255 have been invited to participate at the trial (inpatients *n* 161, outpatients = 94) and thereof 142 patients have provided informed consent (inpatients *n* 75, outpatients = 67). Finally, 132 patients and a further 64 individuals without mental illness participated in the interview, evenly distributed across the three study groups (67 outpatients, 65 inpatients, 64 controls without mental illness). The mean age was 39·6 years (sd = 12·03), and the mean body mass index was 25·8 kg/m^2^ (sd = 4·97). Half of the participants were female and 25 out of 196 participants (12·8 %) had their highest education obtained as the compulsory education. One-quarter of patients (25·8%) were diagnosed with psychosis-related illness (International Classification of Disease-F2) and the others (74·2 %) with affective disorders (International Classification of Disease-F3). All reported characteristics, with exception for gender and age, differed significantly between study groups (see Table 3).

#### Research assistant feedback

Research assistants rated the screening tool as very practical, as it was generally well understood and could be completed in a short time. It was well received by the patients and was compact and yet comprehensive in terms of content (see online Supplementary file).

#### Frequency of nutrition risks being reported

Only seven patients (5·3 %) did not report a nutrition risk, in comparison with 42 (65·6 %) controls. The prevalence for each item listed in the NutriMental screener is presented in Table 4. With the exception of endocrine or metabolic disorders (e.g. diabetes, hyperlipidaemia), all items differed significantly between patients and controls, with higher prevalences in the patient group. Most prevalent issues that required diet-related support were medical conditions (58·3 %), significant changes in weight over the last 6 months (62·9 %) or in appetite over the last month (54·5 %) or changes in weight or appetite since taking a new medication (56·5 % of those taking a new medication). However, all other NutriMental Screener items were also affirmed by at least 10% of the patients.

**Table 4.** NutriMental Screener data from feasibility study in research setting in Switzerland (Numbers and percentages; mean values and standard deviations)

	Outpatients (*n* 67)		Inpatients (*n* 65)		All patients (*n* 132)		Controls (*n* 64)		
	*n*	%	*n*	%	*n*	%	*n*	%	
1. Do you have any of the following conditions requiring dietitian/clinical nutritionist management?	42	62·7 %	35	53·8 %	77	58·3 %	10	15·6 %	< 0·001
• Underweight (< 20·0 kg/m^2^) or overweight (> 30·0 kg/m²)*	9	13·4 %	13	20·0 %	22	16·7 %	3	4·7 %	0·033
• Eating disorder (e.g. anorexia nervosa, bulimia nervosa)	Exclusion criterion within this research study								
• Disorder of gastrointestinal system (e.g. difficulties swallowing, nausea, diarrhea)	8	11·9 %	16	24·6 %	24	18·2 %	4	6·3 %	0·009
• Endocrine or metabolic disorder (e.g. diabetes, hyperlipidaemia)	6	9·0 %	5	7·7 %	11	8·3 %	2	3·1 %	0·445 (Fisher)
• CVD (e.g. stroke)	6	9·0 %	8	12·3 %	14	10·6 %	1	1·6 %	0·050 (Fisher)
• Others (e.g. cancer): ____________________________	27	40·3 %	7	10·8 %	34	25·8 %	1	1·6 %	< 0·001
2. Over the past month, have you had fewer than three defecations (opened bowels) per week?	13	19·4 %	9	13·8 %	22	16·7 %	0	0·0 %	0·001
3. Over the last 6 months, have you the client had any significant changes in weight (loss or gain) without trying?	54	80·6 %	29	44·6 %	83	62·9 %	5	7·8 %	< 0·001
4. In the last month, have you had any significant changes in your appetite or eating behaviour?	34	50·7 %	38	58·5 %	72	54·5 %	9	14·1 %	< 0·001
5. Since taking a new medication, have you had any significant changes in weight (loss or gain) without trying or in your appetite or eating behaviour?	37	60·7 %	28	51·9 %	65	56·5 %	1	14·3 %	< 0·001
Number of participants taking a new medication:	*61*		*54*		*115*		*7*		
6. During the last month, have there been several days where you have experienced strong urges to eat which you are unable to control?	30	44·8 %	25	38·5 %	55	41·7 %	6	9·4 %	< 0·001
7. In the last month, have there been several days when you have been thinking obsessively about food?	18	26·9 %	13	20·0 %	31	23·5 %	0	0·0 %	< 0·001
8. During the past month, have you fasted, vomited or taken laxatives in attempt to control weight?	12	17·9 %	2	3·1 %	14	10·6 %	1	1·6 %	< 0·001 (Fisher)
9. In the last month, did you worry that there would not be enough to eat because of lack of money?	18	26·9 %	16	24·6 %	34	25·8 %	0	0·0 %	< 0·001
10. Would you like support from a dietitian/clinical nutritionist?	34	50·7 %	25	38·5 %	59	44·7 %	4	6·3 %	< 0·001
NutriMental Score*, m (sd)	3·9	1·98	3·0	1·75	3·4	1·91	0·5	0·84	< 0·001 (ANOVA)

* The NutriMental Score corresponds to the number of selected items (without item 10) and ranges from 0 to 9.

In total, one-third (36·4 %) and two-thirds (68·1 %) of patients were screened to be at risk for malnutrition by means of the adapted NRS and the adapted MNA-SF, respectively. But there is no significant difference in the NutriMental score for patients with or without nutrition risk according to traditional risk scores for malnutrition, such as the adapted NRS (*P* = 0·288) or the adapted MNA-SF (*P* = 0·636) (see Table 5).

**Table 5.** Descriptive statistics for the NutriMental score in all patients (*n* 132) (Mean values and standard deviations)

Tool	*n*	NutriMental score		Statistical test
		Mean	sd	
*NRS*				
At-risk (≥ 3 points)	48	3·7	2·06	T = 1·068; *P* = 0·288
Not at-risk (≤ 2 points)	84	3·3	1·82	
*MNA-SF*				
Not at-risk (≥ 12 points)	42	3·5	1·63	T = −0·474; *P* = 0·636
At-risk (≤ 11 points)	90	3·4	2·04 3	
NutriMental Item 10 – Desire for dietary support				
Yes	59	3·6	1·89	T = 1·149; *P* = 0·253
No	73	3·3	1·92	

Almost half of the patients (44·7 %) wished support from a dietitian or clinical nutritionist. Though there was no significant association between the desire for dietary support (NutriMental screener item 10) and the NutriMental score (comprising NutriMental screener items 1–9; *P* = 0·253), there were significant associations with the presence of constipation (NutriMental screener item 2; *P* = 0·015) and significant weight changes in the past 6 months (NutriMental screener item 3; *P* = 0·076).

## Discussion

We developed a multifaceted nutrition-risk screening tool for mental health services through an international, stakeholder engagement approach. This nutrition-risk screening tool, the NutriMental screener, consists of ten items covering frequent nutrition risks experienced by mental health service users that indicate the need for referral to a dietitian or clinical nutritionist. Preliminary testing in a Swiss population group suggests that the tool is feasible to be completed by staff in the presence of mental health service users.

All items of the NutriMental screener were found to be highly relevant for psychiatric patients and more frequently an issue in patients than in healthy controls. The most prevalent risks were under- or overweight, weight changes and appetite changes, often linked to the introduction of a new medication. In line with this, the qualitative interviews from development phase 2 revealed that the mental burden arising from dissatisfaction with their body weight is a key issue for many patients. This leads ultimately to negative connotations, such as stigma and guilt, when talking about their dietary behaviour^(20)^. However, in inpatients, weight loss was also frequently reported^(7)^.

On average, 3·4 nutrition risks were reported for each patient by means of the NutriMental screener. This highlights that patients are exposed to several nutrition risks that cannot be identified by conventional screening tools focusing on single risks only. This assumption is backed up by the fact that no association was found between the NutriMental score and other malnutrition risk scores, such as the adapted NRS and adapted MNA-SF.

One of the strengths of the project is that participatory and evidence-based methods were combined, and representatives of different professional groups from different settings in different countries were involved in the development of the NutriMental screener. The different perspectives included during development suggest that the tool is applicable to mental health services, as reflected in the feedback by research assistants in this study. However, a key limitation is that further implementation studies will be required to understand and address the barriers and challenges when used by mental health clinicians in routine clinical care. The NutriMental screener is now undergoing feasibility testing with mental health clinicians implementing the screener in inpatient and community mental health settings in Australia and in a mental health hospital in Austria.

In this study, almost half of the patients wanted support from a dietitian. This high proportion is not surprising as patients from Austria and Germany reported recently that they often felt left alone with weight-related side effects^(32)^. The desire for dietary support was associated with reporting relevant weight changes in the past 6 months and constipation in the past month, but not with the overall NutriMental score. One explanation might be that patients are not aware of the diverse nutrition risks they are exposed to, maybe as physical health consciousness is limited or of low priority given the mental health burden during acute crises, especially in the inpatient setting^(12,33)^. Mismatches in the number of nutrition risks (NutriMental score) and the desire for dietary support have to be carefully considered in a shared decision-making process^(34)^ to ensure that the scarce resources for dietary support are used wisely. With the aim to develop clinical decision-making aids for care planning based on the NutriMental screener (such as cut-off values), feasibility and clinical implementation are currently being explored in a mixed-methods approach in psychosomatic rehabilitation clinics in Germany^(35)^.

Following the above-mentioned multi-setting feasibility testing in Australia, Austria and Germany, alterations to the NutriMental screener will be made as required, and then formal validation studies will be conducted. These studies will aim to clarify the extent to which the individual questions are sufficient to depict complex problems, such as disordered eating patterns, by comparing them with standardised questionnaires (e.g. the Eating Disorder Inventory^(36)^). In addition, it will be investigated whether the introduction of nutrition-related screening can actually increase the focus on needs in mental health care and thus enable nutritional therapy services to be prescribed in a more targeted and efficient manner.

### Conclusion

There is the potential for the NutriMental Screener to be used as routine screening in various mental health settings, similar to malnutrition screening tools in other hospital sectors. After further validity testing is completed, the NutriMental screener could act as a tool to identify patients ‘at risk’ and allow referral to dietitians for a more comprehensive nutritional assessment. Thus, the implementation of the NutriMental screener in routine care could enable low-threshold access to diet-related support and might be a first step to rectifying the physical-health disparities and life-expectancy gap for people with mental illnesses.

## Supporting information

Mueller-Stierlin et al. supplementary material Mueller-Stierlin et al. supplementary material

## Supplementary material

For supplementary material/s referred to in this article, please visit https://doi.org/10.1017/S0007114524002101
