# Supplementary material for: Feasibility of a multifaceted nutrition-risk screening tool for mental health settings: the NutriMental screener
Source: Br J Nutr. 2024 Oct 29;135(5):559–66. doi: 10.1017/S0007114524002101 (PMC13161525; doi:10.1017/S0007114524002101)
Supplement: Mueller-Stierlin et al. supplementary material [file S0007114524002101sup001.docx]

Supplementary table 1. Initial draft version of the NutriMental screener to be discussed at second workshop


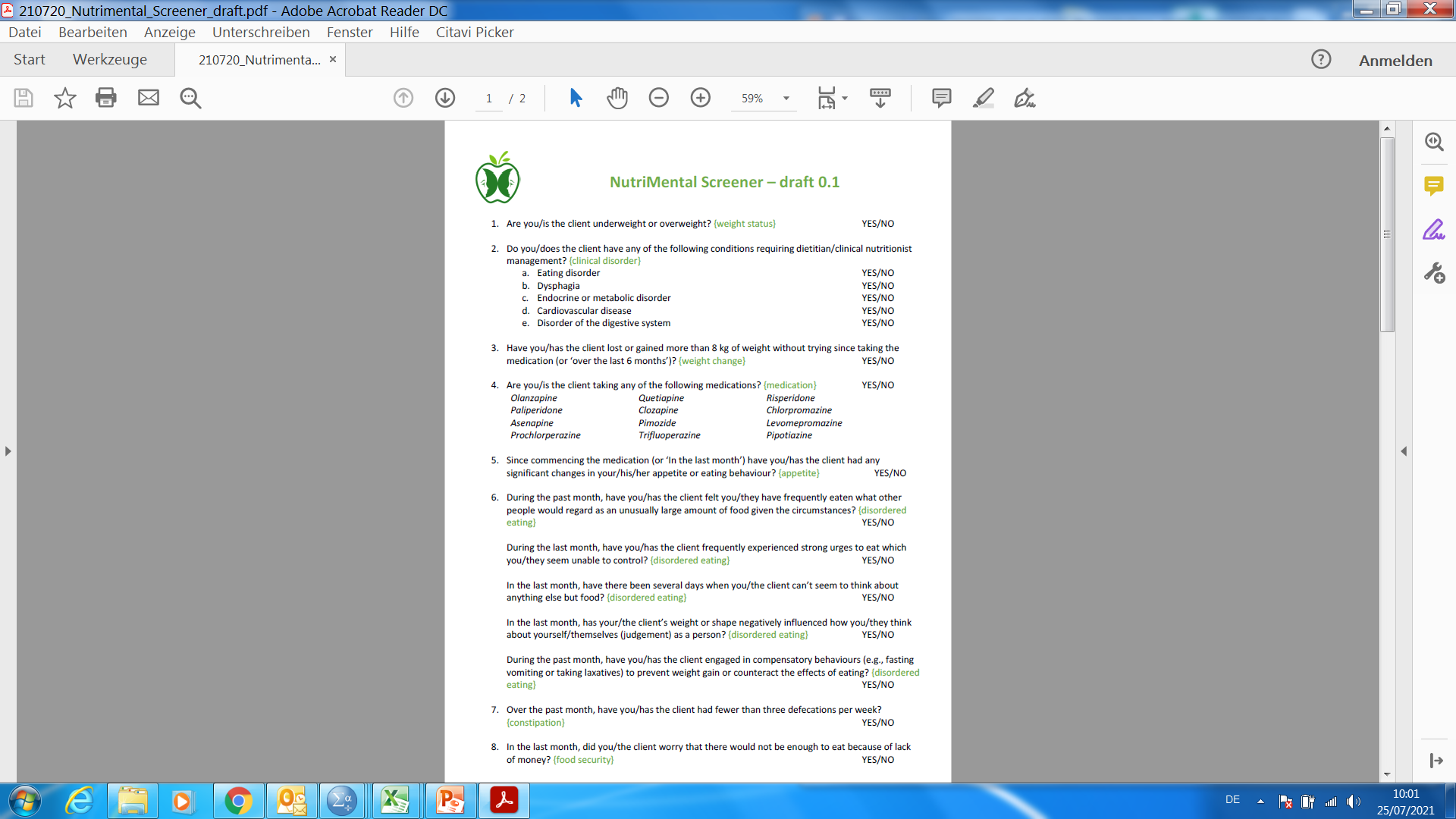

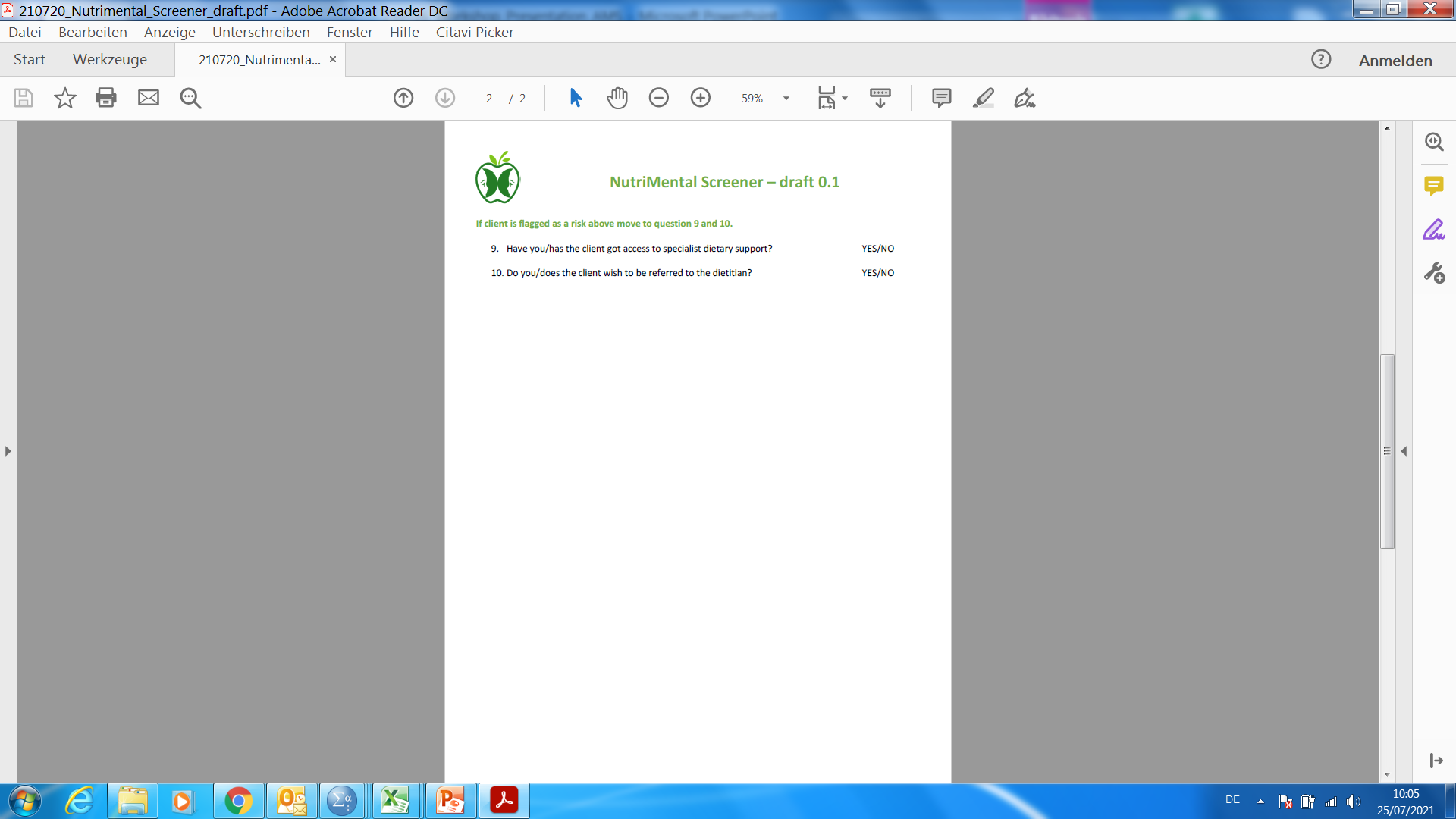


Supplementary table 2. First version of the NutriMental screener to be used for feasibility testing

|  | **NutriMental Screener**  Please, answer all questions to the best of your knowledge. |  |
| --- | --- | --- |
| 1 | Do you have any of the following conditions requiring dietitian/clinical nutritionist management? | □ yes □ no |
| 1.a | underweight (< 20.0 kg / m²) or overweight (> 30.0 kg / m²) | □ yes □ no |
| 1.b | eating disorder (e.g. anorexia nervosa, bulimia nervosa) | □ yes □ no |
| 1.c | disorder of gastrointestinal system (e.g. difficulties swallowing, nausea, diarrhea) | □ yes □ no |
| 1.d | endocrine or metabolic disorder (e.g. diabetes, hyperlipidaemia) | □ yes □ no |
| 1.e | cardiovascular disease (e.g. stroke) | □ yes □ no |
| 1.f | others: ____________________________ (e.g. cancer) | □ yes □ no |
| 2 | Over the past month, have you had fewer than three defecations (opened bowels) per week? | □ yes □ no |
| 3 | Over the last 6 months, have you the client had any significant changes in weight  (loss or gain) without trying? | □ yes □ no |
| 4 | In the last month, have you had any significant changes in your appetite or eating behaviour? | □ yes □ no |
| 5 | Since taking a new medication, have you had any significant changes in weight  (loss or gain) without trying or in your appetite or eating behaviour? | □ yes □ no |
|  | □ Current medication: ___________________________________________________  □ not taking any medication | |
| 6 | During the last month, have there been several days where you have experienced strong urges to eat which you are unable to control? | □ yes □ no |
| 7 | In the last month, have there been several days when you have been thinking obsessively about food? | □ yes □ no |
| 8 | During the past month, have you fasted, vomited or taken laxatives in attempt to control weight? | □ yes □ no |
| 9 | In the last month, did you worry that there would not be enough to eat because of lack of money? | □ yes □ no |
| 10 | Would you like support from a dietitian/clinical nutritionist? | □ yes □ no |

Supplementary table 3. Findings of the feedback questionnaire, completed by research assistants at the end of feasibility testing (n = 5)

| ***Feasibility*** | **Minimum** | **Median** | **Maximum** |
| --- | --- | --- | --- |
| I have been confident this screening based on available terminology and instructions. | I agree | I agree completely | I agree completely |
| Clients were able to understand and respond appropriately to the questions. | I agree | I agree completely | I agree completely |
| Sufficient resources were available allowing me to conduct the screening as part of routine care. | I agree completely | I agree completely | I agree completely |
| ***Acceptance*** | **Minimum** | **Median** | **Maximum** |
| This screening tool contains too many questions. | I strongly disagree | I strongly disagree | I strongly disagree |
| I would perform this screening routinely in a clinical setting. | I am neutral | I agree completely | I agree completely |
| ***Appropriateness*** | **Minimum** | **Median** | **Maximum** |
| The screening targets the needs of our clients. | I agree | I agree | I agree |
| Important nutrition-related issues are missing. | I disagree | I disagree | I am neutral |
| This screening is more suitable than standard malnutrition screenings for use in mental health institutions. | I am neutral | I am neutral | I am neutral |
| This tool has caused me to consider the physical health of clients more. | I agree | I agree completely | I agree completely |
| This screening identified clients in need of assistance with nutrition-related problems. | I am neutral | I agree completely | I agree completely |
| This screening tool is useful for treatment planning. | I agree | I agree completely | I agree completely |
| This screening tool can be used for referral to appropriate professionals, such as dietitians. | I agree | I agree | I agree completely |
